# Tb^3+^-Cleavage Assays Reveal Specific Mg^2+^ Binding Sites Necessary to Pre-fold the *btuB* Riboswitch for AdoCbl Binding

**DOI:** 10.3389/fchem.2017.00010

**Published:** 2017-03-21

**Authors:** Pallavi K. Choudhary, Sofia Gallo, Roland K. O. Sigel

**Affiliations:** Department of Chemistry, University of ZürichZürich, Switzerland

**Keywords:** riboswitch, Tb^3+^, metal ion binding, tertiary interactions, coenzyme B_12_

## Abstract

Riboswitches are RNA elements that bind specific metabolites in order to regulate the gene expression involved in controlling the cellular concentration of the respective molecule or ion. Ligand recognition is mostly facilitated by Mg^2+^ mediated pre-organization of the riboswitch to an active tertiary fold. To predict these specific Mg^2+^ induced tertiary interactions of the *btuB* riboswitch from *E. coli*, we here report Mg^2+^ binding pockets in its aptameric part in both, the ligand-free and the ligand-bound form. An ensemble of weak and strong metal ion binding sites distributed over the entire aptamer was detected by terbium(III) cleavage assays, Tb^3+^ being an established Mg^2+^ mimic. Interestingly many of the M^n+^ (*n* = 2 or 3) binding sites involve conserved bases within the class of coenzyme B_12_-binding riboswitches. Comparison with the published crystal structure of the coenzyme B_12_ riboswitch of *S. thermophilum* aided in identifying a common set of M^n+^ binding sites that might be crucial for tertiary interactions involved in the organization of the aptamer. Our results suggest that M^n+^ binding at strategic locations of the *btuB* riboswitch indeed facilitates the assembly of the binding pocket needed for ligand recognition. Binding of the specific ligand, coenzyme B_12_ (AdoCbl), to the *btuB* aptamer does however not lead to drastic alterations of these M^n+^ binding cores, indicating the lack of a major rearrangement within the three-dimensional structure of the RNA. This finding is strengthened by Tb^3+^ mediated footprints of the riboswitch's structure in its ligand-free and ligand-bound state indicating that AdoCbl indeed induces local changes rather than a global structural rearrangement.

## Introduction

Riboswitches are a class of non-coding RNA that bind small molecules, i.e., metabolites, with high affinity and specificity (Mironov et al., 2002; Nahvi et al., 2002; Winkler et al., 2002). The two functional domains of the riboswitch, the aptamer, and the downstream situated expression platform undergo concomitant structural changes upon ligand binding to the aptamer region (Mandal and Breaker, 2004). This conformational switch ultimately regulates gene expression mainly at the transcriptional or translational level although other regulatory mechanisms have been proposed involving splicing, self-cleavage of the RNA and *trans* regulation (Kubodera et al., 2003; Winkler and Breaker, 2003; Soukup and Soukup, 2004; Winkler et al., 2004; Loh et al., 2009; Bastet et al., 2011).

The *btuB* riboswitch from *E. coli* belongs to the class of coenzyme B_12_ riboswitches recognizing and regulating the pool of coenzyme B_12_ (AdoCbl) inside the cell by altering the expression of the outer membrane B_12_ transporter BtuB (Reynolds et al., 1980; Gudmundsdottir et al., 1988; Nou and Kadner, 2000; Nahvi et al., 2004). The secondary structure of the *btuB* aptamer consists of a four way junction common with the other B_12_ riboswitches and a varying peripheral region (Nahvi et al., 2004). The two recently solved crystal structures of AdoCbl riboswitches from thermophiles have established the role of the four way junction in constituting the ligand binding pocket/the receptor core. The peripheral elements on the other hand further stabilize the binding pocket upon ligand binding and are crucial for specificity toward AdoCbl (Johnson et al., 2012; Peselis and Serganov, 2012).

In many riboswitches the aptamer is pre-organized to facilitate the interaction with its cognate ligand as was observed for the purine, lysine, FMN, SAM I, glucosamine-6 phosphate, and the preQ_1_ binding riboswitches (Hampel and Tinsley, 2006; Klein and Ferré-D'Amaré, 2006; Noeske et al., 2007; Garst et al., 2008; Montange and Batey, 2008; Baird and Ferré-D'Amaré, 2010; Heppell et al., 2011; Suddala et al., 2013). Due to their physiological abundance, divalent metal ions like Mg^2+^ help to assemble the active tertiary structure of the polyanionic RNA not only by shielding the negatively charged sugar-phosphate backbone but also by facilitating specific tertiary interactions within nucleobases (Misra and Draper, 1998; Pyle, 2002; Woodson, 2005). These metal ion interactions are mediated either through an inner sphere or an outer sphere co-ordination where the contacts to the RNA are made directly or via water molecules, respectively (Erat and Sigel, 2011; Schnabl et al., 2011).

Our earlier studies (Choudhary and Sigel, 2014) have given a strong indication that the *btuB* riboswitch is prefolded by Mg^2+^ already in the absence of its ligand AdoCbl. We also observed that AdoCbl does not switch the RNA conformation in the absence of Mg^2+^, indicating the essential role of Mg^2+^ in the pre-organization of the aptamer to enable ligand binding. However, the specific tertiary interactions induced by Mg^2+^ that constitute the ligand-free state of the riboswitch are still not defined. So far there was only little structural insight on the ligand-free fold of the B_12_-riboswitches. All secondary structural models were and still are based on the B_12_-bound state, and also the crystallized coenzyme B_12_ riboswitches represent the ligand-bound structure (Nahvi et al., 2004; Johnson et al., 2012; Peselis and Serganov, 2012). In order to validate the Mg^2+^ aided pre-organization of the *btuB* riboswitch in the absence of AdoCbl it is therefore crucial to locate the specific Mg^2+^ binding sites of this RNA.

A well-established method to map metal ion binding sites in large nucleic acids makes use of lanthanide(III) probes (Ciesiolka et al., 1989; Dorner and Barta, 1999; Sigel et al., 2000; Walter et al., 2000; Kaye et al., 2002; Sigel and Pyle, 2003; Waldsich and Pyle, 2008). Lanthanide(III) ions, specifically Tb^3+^ has been used as the closest mimic for Mg^2+^ due to its similar properties (Sigel et al., 2000; Sigel and Pyle, 2003). The ionic radius of hydrated Tb^3+^ (0.91 Å) is in the same range as hydrated Mg^2+^ (0.71 Å) and both ions have similar preferences for co-ordination to oxygen ligands (Saito and Suga, 2002). It has been shown that Tb^3+^ can replace Mg^2+^ at its specific binding sites without the rupture of the tertiary structure of the RNA (Sigel et al., 2000). Application of Tb^3+^ has the decisive advantage, that this ion can be applied at much lower concentrations and in parallel to Mg^2+^, still yielding a clear cleavage pattern at physiological pH. This is due to the lower p*K*_*a*_ (~7.9) of the Tb^3+^ aqua species [Tb(H_2_O)_*n*_]^3+^ compared to the one of hydrated Mg^2+^ (p*K*_*a*_ ~ 11.4) (Sigel et al., 2000; Harris and Walter, 2003).

Our current studies describe the specific M^*n*+^ (*n* = 2 or 3) binding sites mapped on the *btuB* riboswitch of *E. coli*, both in the ligand-free *btuB* aptamer as well as in the AdoCbl-bound form, in order to establish the role of the Mg^2+^-assisted tertiary interactions for ligand binding. Furthermore, we compared our data to the consensus sequence (Nahvi et al., 2004) as well as to the sequences of the two crystallized AdoCbl riboswitches (Johnson et al., 2012; Peselis and Serganov, 2012) to see if there is a common set of Mg^2+^-mediated tertiary interactions crucial for the organization of the AdoCbl aptamer.

## Materials and methods

### Materials

Nucleoside 5′ triphosphates (ATP, GTP, CTP, and UTP) were purchased from GE Healthcare and Sigma-Aldrich, respectively. Homemade T7 RNA polymerase (Gallo et al., 2005) was used for *in-vitro* RNA transcription. RNase T1 1000 U/μL was purchased from Fermentas and was diluted to 1 U/μL in a buffer containing 50 mM Tris-HCl (pH 7.4) and 50% (v/v) glycerol. Coenzyme B_12_ (Sigma-Aldrich) and anhydrous TbCl_3_ (Sigma-Aldrich) were used without any further purification. Denaturing polyacrylamide gels were prepared using Long Ranger™ gel solution, Lonza, Rockland ME (USA). All the buffers, salt solutions and gel solutions were filtered through 0.2 μm filters. All other chemicals were at least puriss p.a. and were purchased from Sigma-Aldrich. Gels were scanned by Storm860 PhosphoImager and analyzed by ImageQuant software (GE Healthcare).

### Preparation of RNA

The *btuB* RNA was obtained by *in vitro* transcription from the plasmid pPC1 by homemade T7 RNA polymerase (Gallo et al., 2005). The plasmid pPC1 is based on the plasmid pSG2 (Gallo, 2009) that contains the natural 202 nucleotide *btuB* aptamer sequence. In addition, pPC1 contains a newly introduced GGA sequence at the 5′-end of the *btuB* aptamer sequence for efficient transcription (Gallo et al., 2005) and a GAGCUCG sequence at 3′-end stemming from digestion with EcoR1 (Promega). These additional nucleotides still render the aptamer active (Supplementary Figure 5). After *in-vitro* transcription of the plasmid DNA, the *btuB* RNA was purified by 10% denaturing PAGE, electroeluted, precipitated with ethanol and concentrated with Vivaspin concentrator (5000 MWCO). The RNA was stored in water at −20°C.

### Tb^3+^ cleavage reaction

TbCl_3_ stock solutions were prepared as previously described (Sigel et al., 2000). In a total volume of 10 μL, 1 μM of unlabeled RNA along with 10 nM of ^32^P-5′-labeled RNA was denatured at 90°C for 45 s in a reaction buffer containing 25 mM MOPS, pH 7.0 and 100 mM KCl followed by the addition of 20 mM MgCl_2_ and incubation at 37°C for 15 min. For the samples with ligand, AdoCbl was added to the RNA sample folded in 20 mM MgCl_2_ and the samples were further incubated at 37°C for 30 min. For the competition experiments with MgCl_2_, the RNA was folded in various MgCl_2_ concentrations after denaturation and before the addition of TbCl_3_. The cleavage reactions were performed for 1 h on ice. Subsequently quenching, buffer [80% formamide (v/v), 10 mM EDTA] was added to the samples to stop the reaction. The intensity of cleavage products did not change much even when the reaction was carried out for 1.5 and 2 h on ice (data not shown). The samples were precipitated with 2.5 volumes of ethanol, resuspended in 5 μL of formamide loading buffer [80% formamide (v/v), 10 mM EDTA; pH 8.0 at 20°C, 2% Bromophenol blue, 2% Xylene Cyanol] and the cleavage products were separated by 10% denaturing PAGE. RNase T1 ladder and alkaline hydrolysis ladder were prepared as described (Regulski and Breaker, 2008). The relative cleavage intensities of the nucleotides with respect to cleavage intensities at 0 mM Tb^3+^ concentration are calculated as described (Choudhary et al., 2014).

## Results

### Tb^3+^ cleavage reveals specific M^n+^ binding sites of the ligand-free *btuB* riboswitch

**Figure 1:**
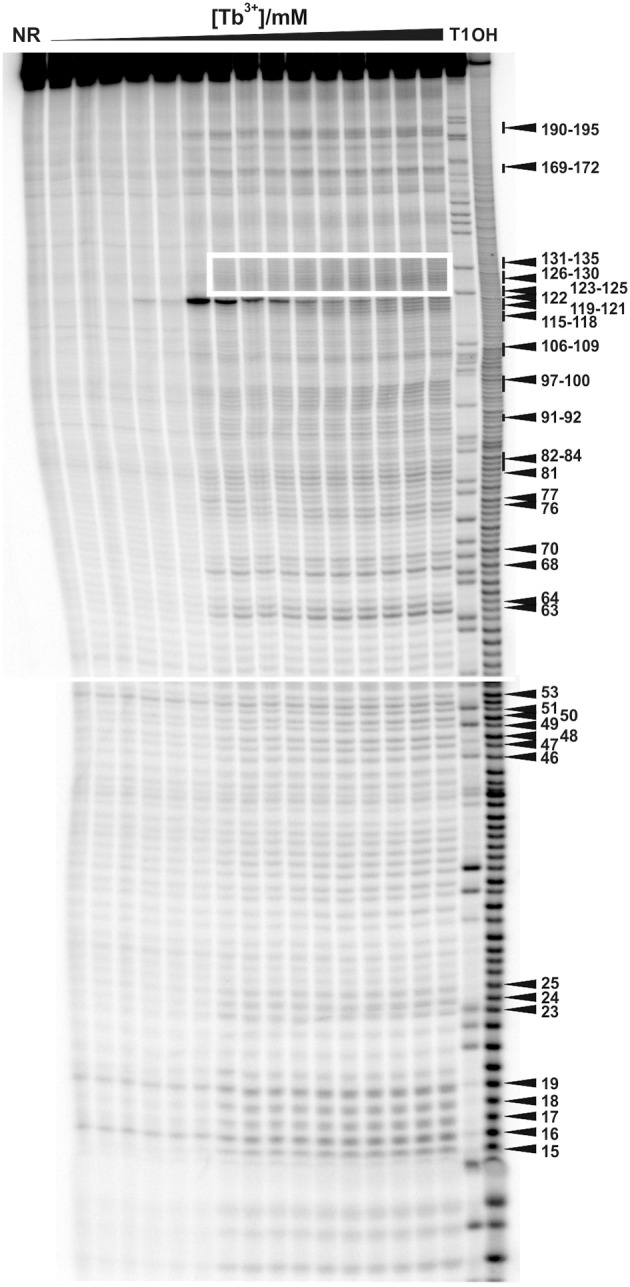
**Tb^3+^ cleavage of the ligand-free and pre-folded *btuB* riboswitch of *E. coli* incubated with increasing concentrations of TbCl_3_ from left to right (0.0, 0.025, 0.05, 0.075, 0.1, 0.25, 0.5, 0.75, 1.0, 2.5, 5.0, 7.5, 10, 15, 20 mM)**. The nucleotides undergoing a distinct cleavage in the presence of TbCl_3_ are indicated on the right side. The white box indicates the supposedly helical region of P12 encompassing nucleotides 123–135. NR, Non-reacted RNA; T1, RNase T1 ladder; OH, alkaline hydrolysis ladder. The Mg^2+^ concentration was held constant at 20 mM.

**Figure 2:**
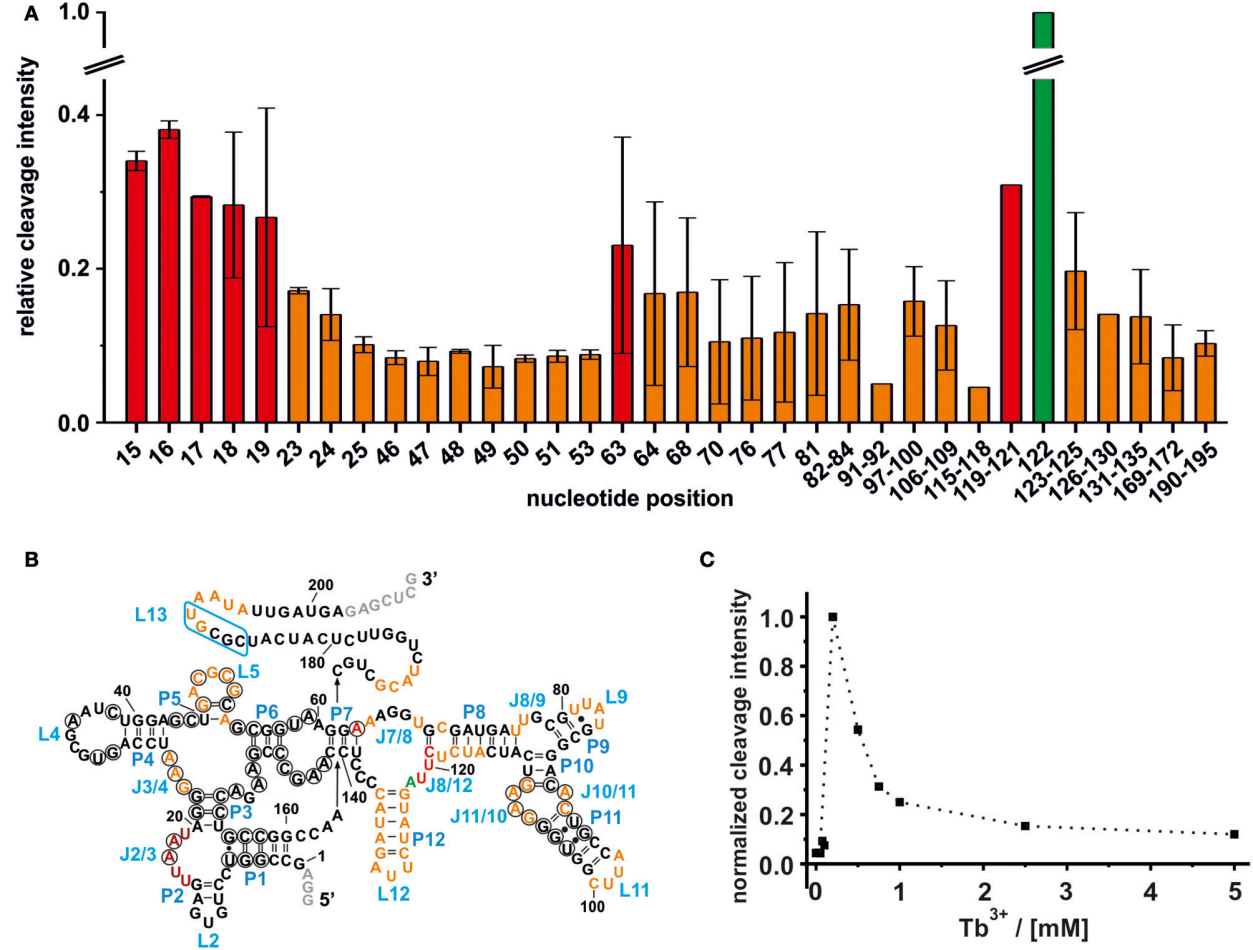
**Mapping of the M^n+^ binding sites on the ligand free *btuB* riboswitch of *E. coli*. (A)** The relative cleavage intensity at the indicated nucleotides at 0.5 mM TbCl_3_ and 20 mM Mg^2+^ by nucleotide is displayed. Sites not showing any intensity change are not displayed. Shown is the average of two gels with the respective range while the cleavage intensity was normalized to the strongest cleavage found at A122. Weak cleavage sites (up to 0.2 relative cleavage intensity) are shown in orange, strong cleavage sites (up to 0.4 relative cleavage intensity) are shown in red, and the strongest cleavage site is shown in green. **(B)** Mapping of the M^n+^ binding sites on the secondary structure of the *btuB* riboswitch. Nucleotides in black circles indicate the conserved bases from the consensus sequence of AdoCbl riboswitches (Nahvi et al., 2004). The blue box indicates the L13 region supposedly undergoing the kissing loop interaction with L5 upon AdoCbl binding. Nucleotides in gray were added to the original *btuB* sequence at both the 3′- and 5′-end to enhance the transcription yield (Choudhary and Sigel, 2014). **(C)** The change in cleavage intensity at A122 (J8/12) as a function of TbCl_3_ concentration derived from one representative experiment.

Tb^3+^ cleavage was carried out with the ligand free *btuB* aptamer pre-folded in 20 mM MgCl_2_ and varying the Tb^3+^ concentration. As shown in Figure 1, Tb^3+^ at concentrations up to 0.1 mM is unable to cleave the RNA indicating that the apparent *K*_D_ of Tb^3+^ to any of the binding sites is higher than 100 μM under these conditions. As soon as the concentration of Tb^3+^ is raised to 0.25 mM, backbone cleavage becomes evident (Figure 1). Usually Tb^3+^ mediated cleavage at micromolar concentration indicates specific metal ion binding sites (Harris and Walter, 2003; Sigel and Pyle, 2003). Hence, the difference in cleavage intensities at 0.5 mM Tb^3+^ compared to the cleavage intensities at 0 mM Tb^3+^ were used to map the specific metal ion binding sites (Figure 2A). This comparison shows that the ligand-free *btuB* aptamer offers M^*n*+^-binding sites of various strengths that are evenly distributed over its whole secondary structure (Figure 2B).

#### Weak M^n+^ binding sites

The intensity of a Tb^3+^ cleavage band depends on the geometry of the coordinating nucleotides within the RNA tertiary fold, i.e., on the accessibility of the 2′-hydroxyl group of their ribose (Harris and Walter, 2003). Weak Tb^3+^ cleavage was categorized as being below 20% cleavage intensity relative to the strongest cleavage observed at A122, and indicates a hindered accessibility due to geometrical constraints. Weak Tb^3+^ cleavage sites on the *btuB* riboswitch are located around the *junctions* J3/4, J7/8, and J8/9, the *loops* L9, L11, and L12, the *internal loop region* of J10/11 and J11/10 and within the *base paired regions* of P5, P8, and P12. Further, weak cleavage sites are situated proximal to nucleotides 169–172, on the kissing loop contributing L5 and around its counterpart at nucleotides190–195 (Figure 2B). Interestingly, some of the cleavage sites at J3/4, P5/L5, and at the internal loop region J11/10 and J10/11 involve nucleotides of conserved regions in the consensus sequence of the coenzyme B_12_ riboswitches (Nahvi et al., 2004). Comparison with the published crystal structures of the coenzyme B_12_ riboswitches from thermophiles (Johnson et al., 2012; Peselis and Serganov, 2012) indicates that the conserved nucleotides at J11/10 contact the nucleotides from the junction J6/3 helping to create one of the coaxial stems of the receptor core. Also metal ion binding at L5 might be important for the observed kissing loop (KL) interaction between L5 and L13 (Johnson et al., 2012; Peselis and Serganov, 2012), while metal ion binding at J3/4 could assist its supposed tertiary interactions to J6/3 and P3 (Peselis and Serganov, 2012).

#### Strong M^n+^ binding sites

The regions J2/3 and J8/12 as well as A63 situated in P7 harbor strong Tb^3+^ cleavage sites involving also the conserved nucleotides at J2/3 and A63 (Figure 2B). Metal ion binding at J2/3 could facilitate the supposed tertiary interactions to P1 as reported for the *S. thermophilum* AdoCbl riboswitch (Peselis and Serganov, 2012). Although strong cleavage is clustered at the above-mentioned sites, we want to draw the attention to the helical region of P12, which is just next to the strongest cleavage site found at A122 (Figures 1, 2B). Even though the nucleotides of P12 are proposed to undergo base pairing in the ligand-bound state (Nahvi et al., 2004) hampering Tb^3+^ accessibility, our experiments show partial cleavage in this region. The region P12 therefore appears to be at least to some extent in a dynamic or non-fully canonical conformation accessible for Tb^3+^ binding when in the ligand-free state. As stated before, nucleotide A122, which exhibits the strongest cleavage of all nucleotides, is situated next to this region of moderate M^*n*+^ binding. The cleavage at nucleotide A122 decreases with increasing Tb^3+^ concentration, a trend completely opposite of any of the other cleavage sites (Figure 2C). We suggest that in contrast to the other sites, the geometry at A122 is altered at high Tb^3+^ concentrations, possibly by additional Tb^3+^ binding close-by, thereby inhibiting the cleavage.

Besides stem P12, all other proposed helical regions of the *btuB* aptamer were found to be unaffected from Tb^3+^-cleavage confirming their helical state also in the ligand-free form of the riboswitch. The lack of cleavage does however not exclude their involvement in metal ion binding since the helical regions often bind metal ions in the major groove without direct access to the 2′-hydroxyl group (Sigel et al., 2000), and therefore metal ion binding sites in such regions remain undetected.

#### Confirmation by competition experiments between Tb^3+^ and Mg^2+^

**Figure 3:**
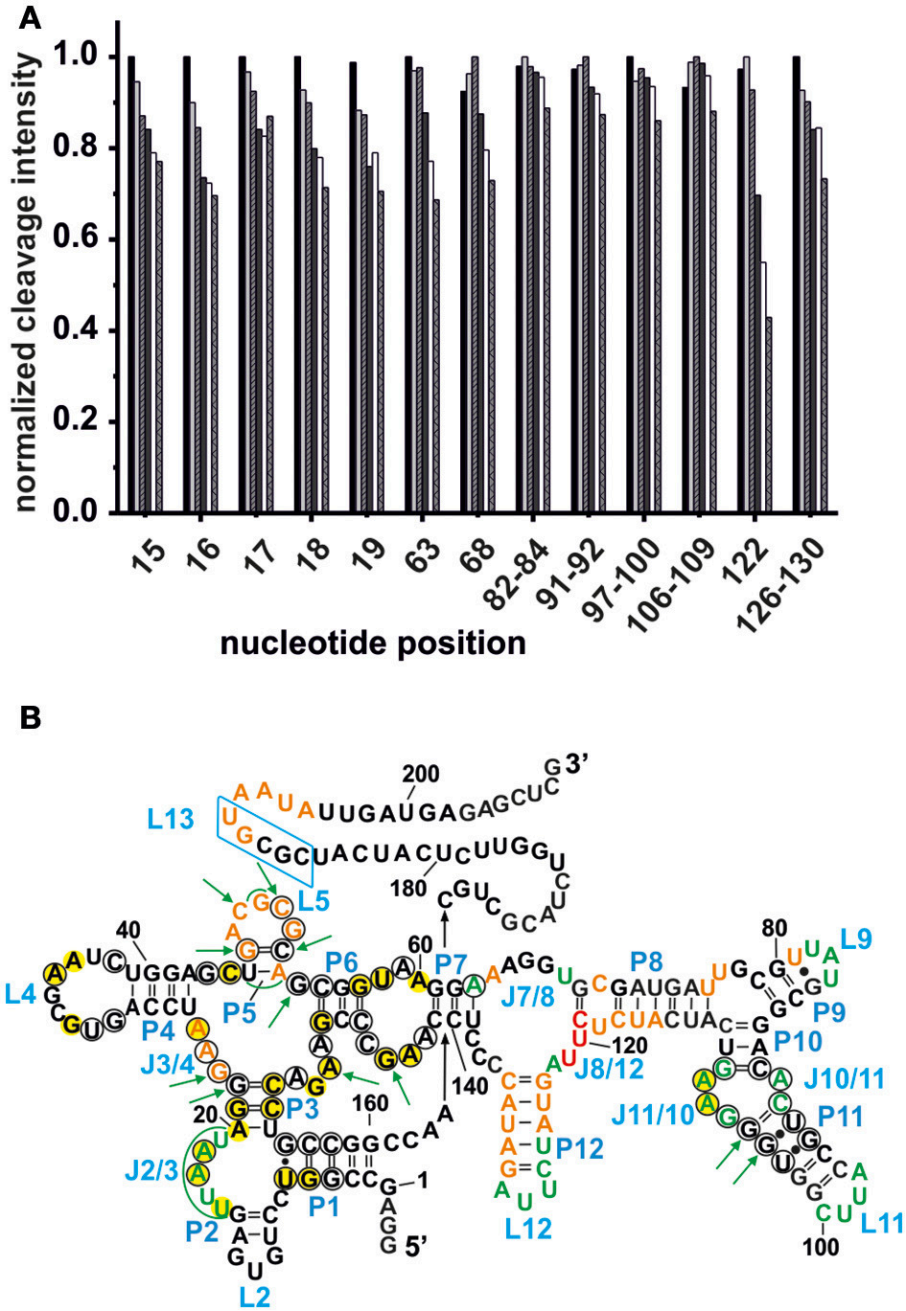
**Competition between Mg^2+^ and Tb^3+^ for the identification of specific M^n+^ binding sites. (A)** The decrease in cleavage intensity at the mapped sites is shown as a function of Mg^2+^ while Tb^3+^ was held constant at 500 μM. The Mg^2+^ concentration increases from 20 to 90 mM (from left-to-right). **(B)** Comparison of the metal ion binding sites of the AdoCbl riboswitches from *E. coli* and *S. thermophilum* (Peselis and Serganov, 2012). Shown is the proposed secondary structure of the *btuB* riboswitch with circled nucleotides representing the conserved bases in the consensus sequence of AdoCbl riboswitches (Nahvi et al., 2004). Green nucleotides correspond to specific Mg^2+^ binding sites of the *btuB* riboswitch. The nucleotides marked by green arrows indicate the metal ion binding sites at conserved nucleotides as found in the *S. thermophilum* AdoCbl riboswitch while areas marked in green signal possible metal ion binding sites at non-conserved nucleotides. Bases marked in yellow could be involved in tertiary interactions as comparison with the *S. thermophilum* riboswitch suggests (Peselis and Serganov, 2012). The blue box indicates the L13 region supposedly undergoing the kissing loop interaction with L5 upon AdoCbl binding. This tertiary interaction is not visible in the crystallized riboswitch from *S. thermophilum* since it was truncated after P1. Orange and red nucleotides were shown to provide weak and strong Tb(III) binding sites respectively (see Figure 2).

Competing Tb^3+^ with an increasing Mg^2+^ concentration results in the decrease of Tb^3+^ cleavage intensity, ideally at nucleotides involved in Mg^2+^ binding, but also in general due to better charge screening by the increased M^2+^ concentration (Harris and Walter, 2003). Hence, not only specific metal ion binding sites are confirmed but also sites of non-specific Tb^3+^ binding are eliminated. In competition experiments with 500 μM Tb^3+^ and increasing concentrations of Mg^2+^, we observed a general decrease in the cleavage intensities at the mapped metal ion binding sites (Figure 3A and Supplementary Figure 1). In the absence of Mg^2+^, no specific Tb^3+^ cleavage is observed, as the riboswitch is apparently unfolded, not reaching a distinct three-dimensional structure. In the presence of Mg^2+^, i.e., when the riboswitch is folded correctly, nucleotides 82–84, 91–92, 97–100, and 106–109 exhibit a less pronounced competition with Tb^3+^ compared to the other sites, probably due to a smaller affinity toward Mg^2+^ ions (Figure 3A). These nucleotides belong to the peripheral structural elements (L9, J10/11, P11, J11/10) of the aptamer and in spite their accessibility as proposed by the comparison to the two solved crystal structures, this region shows a lower affinity to Mg^2+^ than the other, probably very structured part of the riboswitch. Interestingly, at all Mg^2+^ concentrations studied (20–100 mM), nucleotide A122 always exhibits the strongest cleavage by Tb^3+^ compared to the other mapped nucleotides. The evident strong cleavage as well as the distinctive decrease in cleavage intensity with increasing Mg^2+^ concentration suggests a strong Mg^2+^ binding site at or close to A122. The competition experiments thus confirm to a large extent the mapped metal ion binding sites as shown in Figures 1, 2.

### The relation between conserved nucleotides, metal ion binding, and tertiary interactions

In each class of riboswitches, the aptameric moiety is higher conserved than the expression platform ensuring a conserved three dimensional structure of the aptamer by tertiary contacts between conserved residues (Roth and Breaker, 2009). Finding a correlation between these conserved nucleotides and their affinity toward Mg^2+^ would provide a general scheme of important Mg^2+^ mediated tertiary interactions among different coenzyme B_12_ riboswitches. We therefore compared the metal ion binding behavior of the *btuB* riboswitch of *E. coli* to the crystallized AdoCbl riboswitch from *S. thermophilum* (Figure 3B) (Peselis and Serganov, 2012). Except for the two additional peripheral stems P9 and P12, the *btuB* riboswitch has a similar secondary structure not only as the *S. thermophilum* one, but also as the second crystallized AdoCbl riboswitch from *T. tengcongensis*. While the crystallized aptamer of the *T. tengcongensis* riboswitch includes stem P13 involved in the L5/L13 kissing-loop interaction, the crystallized construct of the *S. thermophilum* riboswitch was shortened on this site (Johnson et al., 2012; Peselis and Serganov, 2012). Nevertheless, it is highly likely that all three riboswitches share a respective tertiary structure of the aptamer region (Nahvi et al., 2004; Johnson et al., 2012; Peselis and Serganov, 2012).

The crystal structure of the AdoCbl riboswitch from *S. thermophilum* solved at 3.05 Å resolution (PDB ID: 4GXY; Peselis and Serganov, 2012) reveals two Mg^2+^ ions (Mg A310 and Mg A311) and seven iridium(III) hexammine complexes. The contact maps for the seven iridium(III) complexes indicate hydrogen bonds not only with O4 and O5 of pyrimidines as well as O6 and N7 of guanines but also with oxygen atoms from the phosphate backbone (Table 1). Since the contact maps for the two remaining Mg^2+^ ions are missing, we applied MINAS (Schnabl et al., 2011) to look for the nearest contacting nucleobases (Table 2). Presumably both Mg^2+^ ions interact with the predicted bases via outer sphere co-ordination since the distances of the contacts are more than 2.7 Å (Supplementary Figure 2 and Supplementary Table 1). One of the Mg^2+^ (A310) contacts a guanine quartet at L8 and P8 (Supplementary Figure 2A) whereas the other Mg^2+^ (A311) is placed in proximity to L5 (Supplementary Figure 2B). Interestingly, out of the 25 residues involved in metal ion binding in the crystal structure, 13 were found to be conserved in the consensus sequence of coenzyme B_12_ riboswitches (Nahvi et al., 2004). Furthermore, the binding of iridium(III) hexammine, to most non-conserved residues, namely to U34, U35, U38, G64, C72, U100, G101, G103, C104, involves the adjacent vicinity to the conserved bases at J2/3, J4/5, P5, and J10/11 (Peselis and Serganov, 2012).

**Table 1:** **Contact map for the ammine ligands (N1–N6) of the iridium(III) hexammine (IRI A303–A309) in the crystal structure of the AdoCbl riboswitch (*S. thermophilum*) (Peselis and Serganov, 2012)**.

**NH_3_**	**IRI (A303)**			**IRI (A304)**			**IRI (A305)**		**IRI (A306)**			**IRI (A307)**			**IRI (A308)**				**IRI (A309)**		
N1													OP1						O6		
N2		O4	OP1/O5			OP1		OP1			OP2						OP2				OP1
N3	O4	O4																N7/OP2			
N4				OP1	OP1/O5				N7/O6			OP1/O5/OP2	OP1		OP1	OP2			O6	O6	
N5						OP1			O6				OP1	OP2							
N6			OP1/O5				OP1			OP2								OP2		O6	
Nucleo-base	U	U	A	G	G^*^	C^*^	U	G	G^*^	C^*^	C^*^	C	A^*^	G^*^	G	C	G^*^	G^*^	G^*^	G^*^	A^*^
	34	35	38	64	65	67	100	101	65	68	69	72	73	151	103	104	122	123	41	42	157
Region	J2/3			L5			J8/10		L5			J5/6; J7/6			J10/11; J11/10				J3/4; J6/3		

*Conserved nucleotides are marked by an asterisk. The conserved A157 (underlined) was shown to interact with the adenine base of the bound AdoCbl*.

**Table 2:** **Outer sphere coordination for Mg^2+^ ions in the crystal structure of the AdoCbl riboswitch (*S. thermophilum*; Peselis and Serganov, 2012) as calculated by MINAS (Schnabl et al., 2011)**.

**Ligand**	**Mg^2+^ (A310)**				**Mg^2+^ (A311)**	
O2′					+	
O4′				+		
O6	+	+	+			
N7		+	+	+		
OP1				+		+
OP2				+		
Nucleo-base	G96	G97	G98	G130	G65^*^	U66
Region	J8/10; P8				P5/L5	

*Numbering is according to the crystal structure and the conserved G65 is marked by an asterisk*.

Comparison of the specific Mg^2+^ binding sites of the ligand-free *btuB* riboswitch with the metal binding sites of the *S. thermophilum* riboswitch crystallized in its AdoCbl-bound form (Peselis and Serganov, 2012) show a good correlation at J2/3 and J11/10 (Figure 3B). Interestingly, these two regions involve conserved nucleotides potentially undergoing crucial tertiary contacts as found for the *S. thermophilum* riboswitch (Peselis and Serganov, 2012). It is therefore highly probable that the long range interaction of J11/10 to J5/6 and J6/3 as well as the P3-P1 stack are already formed in the ligand-free form of the riboswitch and that they are facilitated by Mg^2+^ binding. Other metal binding sites of the *S. thermophilum* riboswitch agree with some of the weaker metal binding sites found by Tb(III) cleavage, namely at J3/4 and P5/L5.

### M^2+^ binding sites in the switched aptamer conformation

The *btuB* riboswitch is known to undergo a conformational change upon AdoCbl-binding mainly through the proposed pseudoknot formation between L5 and L13 disrupting thereby the anti-terminator stem (Nahvi et al., 2002; Gallo et al., 2008). We attempted to visualize this conformational change in terms of M^n+^ binding properties. For this we compared its Mg^2+^ binding sites described above with the results of the ligand-bound aptamer. The *btuB* riboswitch was prefolded in 20 mM MgCl_2_ and then incubated with AdoCbl for 30 min before addition of 0.5 mM Tb^3+^. The incubation time of 30 min with AdoCbl was adequate for a structural switch of the RNA (Supplementary Figure 3), as indeed the conformational switch appears instantaneously upon addition of AdoCbl.

**Figure 4:**
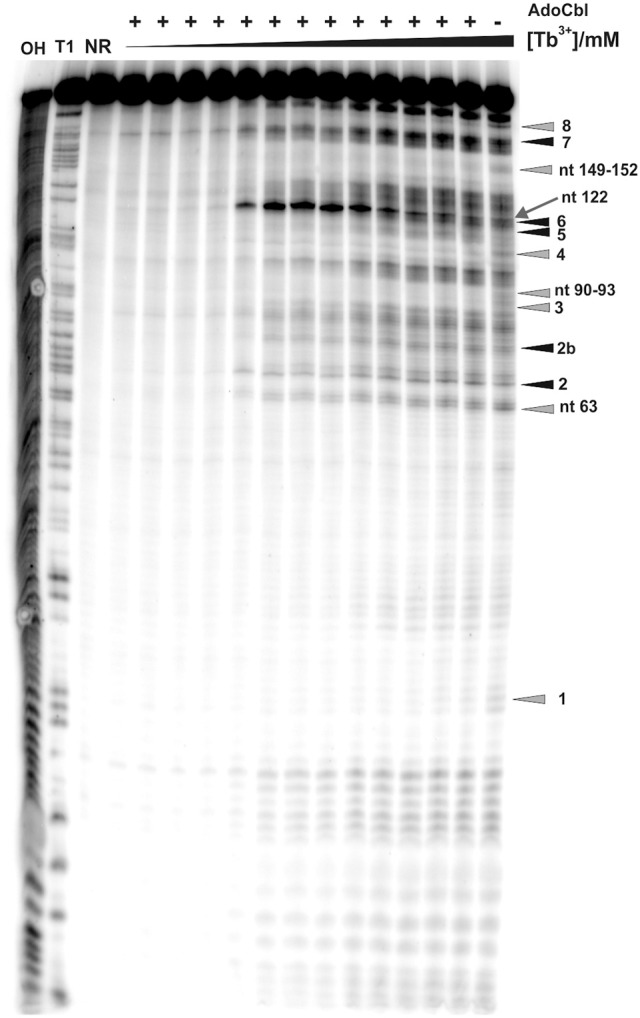
**Tb^3+^ cleavage of the ligand bound *btuB* riboswitch**. The RNA was prefolded in the presence of 100 μM AdoCbl and then incubated with increasing concentrations of TbCl_3_ (from left to right, 0.0, 0.025, 0.05, 0.1, 0.25, 0.5, 0.75, 1.0, 2.5, 5.0, 10, 15, 20 mM). The arrow shows nucleotide A122 displaying the strongest cleavage of all sites within the *btuB* riboswitch. Triangles in gray and black indicate the nucleotides with decreased and increased cleavage in the presence of AdoCbl (Supplementary Figure 4). The cleavage patterns of the two lanes on the right [20 mM Tb(III), ± AdoCbl] were used for the structural footprints shown in Figure 6. The numbered cleavage bands indicated on the right side correspond to the previously described in-line probing bands (Gallo et al., 2008). OH, Alkaline hydrolysis ladder; T1, RNase T1 ladder; NR, non-reacted RNA.

**Figure 5:**
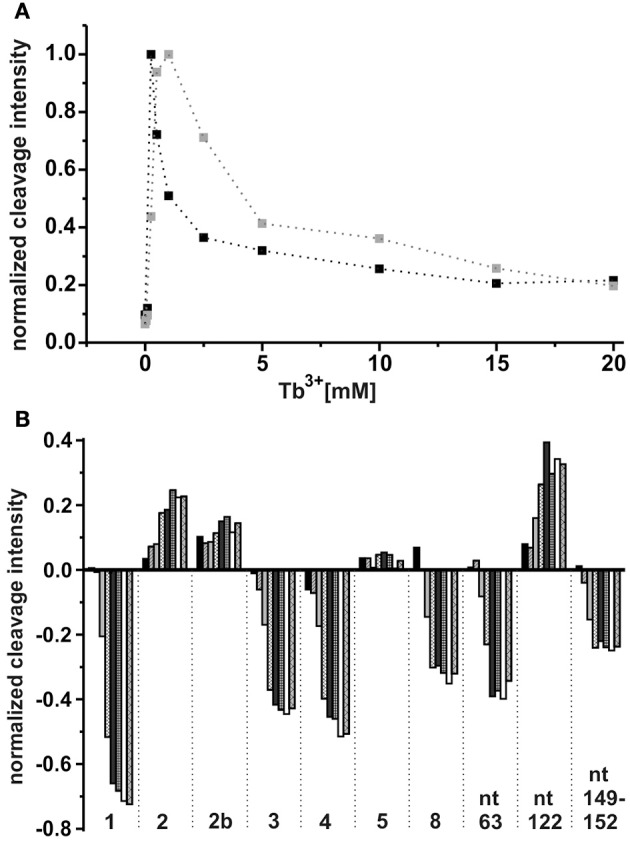
**Tb^3+^ dependent changes in the ligand bound conformation of the *btuB* riboswitch. (A)** TbCl_3_ concentration dependent changes at nucleotide A122 in the absence (black squares) and presence (gray squares) of 100 μM AdoCbl. The data points are connected by dotted lines for better visibility. **(B)** AdoCbl concentration dependent changes at the indicated nucleotides. The RNA was incubated with AdoCbl (0.05, 0.1, 0.5, 1, 5, 10, 50, 100 μM) prior to cleavage with 0.5 mM TbCl_3_.

As already observed for the ligand-free RNA, cleavage of the AdoCbl-bound aptamer was again evident at Tb^3+^-concentrations higher than 0.25 mM (Figure 4). Interestingly, at Tb^3+^ concentrations between 0.25 and 1 mM, the strongest cleavage is again observed at nucleotide A122 (J8/12). However, in the presence of AdoCbl, the cleavage intensity of A122 achieves its maximum only at 1 mM Tb^3+^ and not at 0.25 mM as in the absence of the ligand (Figure 5A), showing a decreased affinity to M^n+^ at this site upon ligand-binding. The overall cleavage pattern for the switched RNA (+AdoCbl) at higher Tb^3+^ concentrations (from 2.5 to 20 mM) remains the same as in the absence of AdoCbl except for the sites modulated by AdoCbl (Sites 1–8). Sites 1 (G23), 3 (G87), 4 (G106), and 8 (U183) undergo relative decrease in cleavage whereas sites 2 (U68), 5 (U110), and 7 (U167) exhibit increase in the cleavage intensity as expected (Figure 5B; Nahvi et al., 2002). However, site 2b (U77) displays an increase in cleavage intensity contrary to observation from the in-line probing experiments (Gallo et al., 2008). The AdoCbl dependent increase in the cleavage at site 2b is confirmed from the experiment where the *btuB* riboswitch was incubated with varying AdoCbl concentrations prior to cleavage by 0.5 mM Tb^3+^ (Supplementary Figure 4). This difference between in-line probing and Tb^3+^ mediated cleavage is probably due to the minor differences in binding between the two metal ions (Sigel and Pyle, 2003).

There is a marked decrease in the cleavage intensity at nucleotides 63 (P7), 90–93 (P10–P11), and 149–152 (J6/3) along with the earlier reported sites modulated by AdoCbl (Figures 4, 5B). This could be due to AdoCbl induced tertiary interactions between P10–P11 and J6/3 in the *btuB* riboswitch similar to the one observed in the two reported crystal structures of AdoCbl riboswitches (Johnson et al., 2012; Peselis and Serganov, 2012).

### Footprints of the secondary and tertiary structure of the *btuB* riboswitch

Besides being a probe for Mg^2+^ binding sites, Tb^3+^ can also be used to investigate the native conformation of RNAs (Harris et al., 2004). At high millimolar concentration Tb^3+^ cleaves RNA additionally in a sequence-independent manner producing a foot printing pattern of single-stranded and non-Watson-Crick base paired elements (Sigel and Pyle, 2003; Harris et al., 2004). We compared the cleavage pattern of the *btuB* riboswitch at 20 mM Tb^3+^ in the absence and presence of AdoCbl (Figure 4, lanes 16 and 17) to get an insight into the arrangement of the riboswitch domains at native pH.

**Figure 6:**
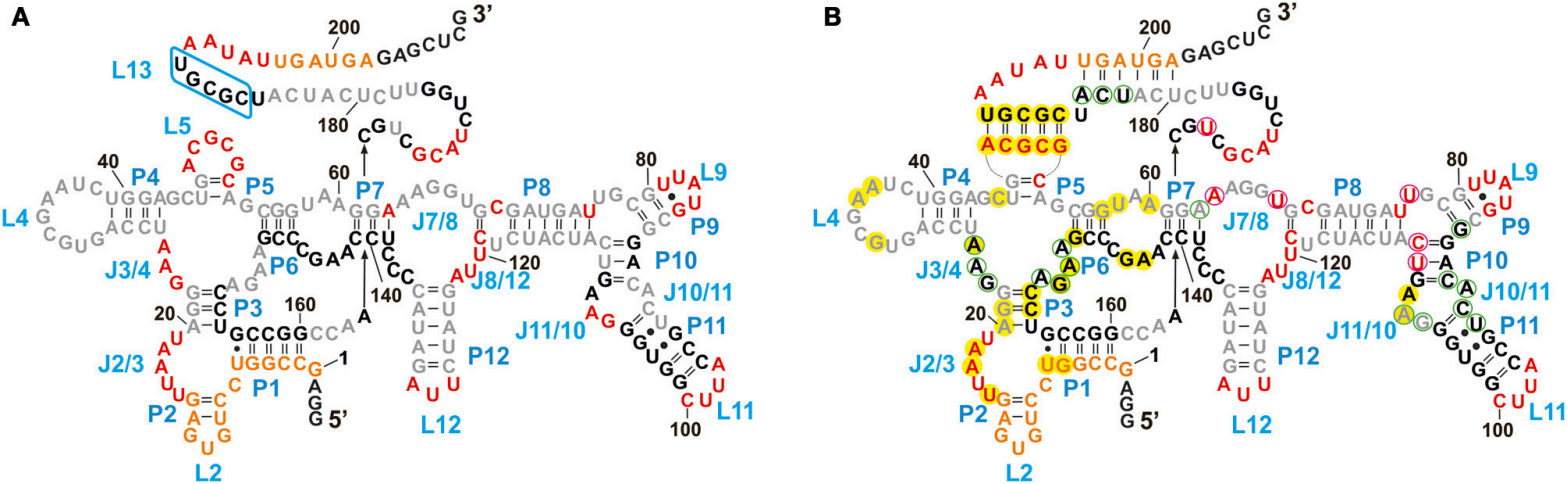
**Structural footprint of the *btuB* aptamer obtained by Tb^3+^ cleavage in the absence (A)** and presence **(B)** of AdoCbl. Nucleotides undergoing strong cleavage in the presence of 20 mM TbCl_3_ are shown in red, the ones with intermediate cleavage are marked in orange. Partially protected nucleotides are shown in gray and completely protected nucleotides are in black. Nucleotides circled in magenta and light green indicate an increased or decreased cleavage intensity, respectively, in the presence of 100 μM AdoCbl and 20 mM Tb(III). Bases marked in yellow could be involved in tertiary interactions as comparison with the *S. thermophilum* riboswitch suggests (Peselis and Serganov, 2012).

In the absence of AdoCbl (Figure 6A), the *strongly cleaved* regions belong to the proposed single stranded regions. These regions mainly involve the junctions (J2/3, J3/4, L5, J7/8, J8/12) and the loops (L9, L11, L12) along with the nucleotides 169–172 and 192–196 surrounding stem P13. The other proposed single stranded regions of the riboswitch, L4, J6/7, J7/8, and nucleotides 177–184 (surrounding stem P13) display relative protection from cleavage. For L4 and J6/7 this indicates their tertiary contact, fortifying the structural similarity of the *btuB* riboswitch to the other two crystallized AdoCbl riboswitches. The protection of the other two regions could be due to surface occlusion within the folded RNA, a finding however not supported by the two crystal structures (Johnson et al., 2012; Peselis and Serganov, 2012).

We observed a *marked protection* from cleavage at the conserved B_12_ box (140–160), P9–P11, J10/11, and J10/11 as well as at the regions encompassing nucleotides 164–168, 173–176, and 186–190. Out of these regions, only a part of the B_12_ box (140–141, 146–148, 153–160) and the peripheral face of the aptamer (at P8–P11) include proposed helical regions leaving the secluded position within the inner core the only explanation for the protection of the other parts. Apart from P9, which is missing in the two AdoCbl riboswitches from thermophiles, and of J3/13 (161–179), which is missing or not defined in the crystal structures, all of these protected areas match very well with the inner core as seen for the two crystallized AdoCbl riboswitches. Clear protection from cleavage is further evident for the regions encompassing nucleotides 20–22 and 26–62 that presumably constitute a part of the receptor core, again as seen for the crystallized AdoCbl riboswitches (Johnson et al., 2012; Peselis and Serganov, 2012).

Upon addition of AdoCbl, the overall global conformation of the aptamer appears similar to the one in the absence of the ligand except for the sites reported to be modulated by AdoCbl (Figure 6B).

## Discussion

Depending on the riboswitch class, the correct pre-organization of the aptameric part is a crucial step for ligand recognition and binding (Montange and Batey, 2008; Baird and Ferré-D'Amaré, 2010). Our earlier studies suggest that this fact is also true for the *btuB* riboswitch from *E. coli* to allow an interaction with its ligand AdoCbl (Choudhary and Sigel, 2014). The formation of this so-called binding-competent fold is mediated by Mg^2+^ binding to specific sites and thus facilitating tertiary interactions within the RNAs tertiary fold. The detection of these binding sites combined with the comparison with a riboswitch of similar structure, in our case the AdoCbl-sensing riboswitch of *S. thermophilum* (Peselis and Serganov, 2012), allowed for a quite adequate prediction of the *btuB* riboswitch tertiary structure. Furthermore, we compared the Mg^2+^ binding sites within the ligand-free and ligand-bound aptamer to reveal structural differences and to demonstrate if indeed there is a global rearrangement upon AdoCbl binding or only locally restricted structural changes.

### M^n+^ binding and conserved tertiary contacts in AdoCbl riboswitches

Mapping the M^n+^ binding sites of the *btuB* riboswitch we observed metal ion binding at/around a set of conserved residues (J2/3, J3/4, P5, L5, P7, J10/11, P11, and J11/10). Interestingly, these regions are very likely to undergo tertiary interactions crucial for the aptamer active structure, as was revealed by structural comparison with the two crystallized AdoCbl riboswitches from thermophiles (Johnson et al., 2012; Peselis and Serganov, 2012). Furthermore, the M^n+^ binding sites of the *btuB* riboswitch show a partial match with the $\text{Ir}{\left({\text{NH}}_{3}\right)}_{6}^{3+}$ binding sites of the *S. thermophilum* riboswitch (Peselis and Serganov, 2012). We therefore propose a common set of Mg^2+^ mediated tertiary contacts for the aptameric part of the AdoCbl-binding subclass of the B_12_-riboswitches.

### Tertiary structure proposal for the *btuB* riboswitch

Both structures of the AdoCbl riboswitches crystallized in their ligand-bound form revealed complex tertiary structural elements such as coaxial stems (P3–P6 and P4–P6), a kissing loop (L5 and L13), a T loop-PK domain interaction between L4 and J6-7/J7-6 as well as a long range tertiary interaction between J10/11 and J6/3 (Johnson et al., 2012; Peselis and Serganov, 2012). In addition to these common features, the structure from *S. thermophilum* included a minor groove interaction of J2/3 with P1 and a zipper formation between J3/4, J6/3 and P3 (16). The formation of tertiary motifs like coaxial stems, kissing loops, and pseudoknots is largely dependent on Mg^2+^ for stabilization (Batey et al., 1999). The evident correlation in M^n+^ binding sites of the *btuB* riboswitch to the two structurally known AdoCbl riboswitches leads to the conclusion that these three riboswitches share a corresponding tertiary fold within their aptameric part.

The observed cleavage pattern at stem P12, missing in the two crystallized riboswitches, indicates a partial dynamic geometry in this region. The biochemical data for AdoCbl riboswitches with and without stem P12 does not correlate to the affinity for AdoCbl (Nahvi et al., 2004). Therefore, we suggest that stem P12 is involved in stabilizing the overall binding pocket but may not necessarily contribute toward the specificity to AdoCbl. Located at the peripheral region of the *btuB* riboswitch is nucleotide A122 that appears to have the most suited geometry for Tb^3+^ binding. The strong cleavage at A122 is evident at low micromolar Tb^3+^ concentrations in both the ligand-free and the ligand-bound form. A122 is located at the J8/12 region of the *btuB* aptamer and since both of the crystallized AdoCbl riboswitches (Johnson et al., 2012; Peselis and Serganov, 2012) lack this peripheral part, it is difficult at present to predict the role of A122 in the overall structure of the *btuB* riboswitch of *E. coli*.

### Local changes upon AdoCbl binding are observed in the *btuB* riboswitch

Our results show similar Tb^3+^ coordination in the ligand-free and ligand-bound forms of the *btuB* riboswitch of *E. coli*. The mapping experiments revealed that the crucial tertiary interactions involving the T loop pseudoknot, the long range interaction of J11/10–J5/6 and J6/3 and the KL-interaction supposed to form upon AdoCbl-binding are already formed in the absence of AdoCbl. Therefore, the binding pocket of the aptamer seems to be highly pre-organized by M^n+^ already in the ligand-free form to allow optimal binding to AdoCbl. This is in strong agreement with our earlier studies (Choudhary and Sigel, 2014) indicating that the *btuB* riboswitch cannot be switched by AdoCbl in the absence of Mg^2+^.

This pre-assembly of the ligand binding pocket solely by Mg^2+^ is not peculiar for the *btuB* riboswitch but has been observed for the adenine riboswitch, the glmS ribozyme and for the lysine binding riboswitches (Garst et al., 2008; Baird and Ferré-D'Amaré, 2010; Fiegland et al., 2012). Mg^2+^ mediated pre-compaction of the RNA in the absence of the ligand has been further reported by SAXS studies for the glycine, the c-di-GMP, the TPP, the lysine, and the SAM I riboswitches (Baird and Ferré-D'Amaré, 2010). Therefore, Mg^2+^ mediated pre-organization of RNA to achieve an active tertiary structure of the ligand-free aptamer seems to be a common phenomenon in riboswitches and probably holds true for the AdoCbl riboswitches as well.

While the *btuB* riboswitch appears not to undergo a drastic structural change upon AdoCbl binding, two distinct local changes take place: First, similar intensity changes upon AdoCbl addition are observed in Tb^3+^ cleavage experiments as reported by in-line probing in earlier studies (Nahvi et al., 2002; Gallo et al., 2008). Second, the observed M^n+^ binding to the two conserved adenosine residues of J11/10 upon AdoCbl binding which might aid its interaction with J6/3.

To summarize, we propose the presence of specific M^n+^ binding sites and the pre-organization of the *btuB* aptamer in its ligand-free form encompassing M^n+^ mediated tertiary interactions within the aptamer. These tertiary interactions could be common for all members of the AdoCbl-binding B_12_ riboswitches in order to facilitate the interaction with their complex ligand, AdoCbl. A pre-organized binding pocket benefits the riboswitch not only with a rapid recognition of the ligand but also with the prompt transmission of the regulatory signal (Baird and Ferré-D'Amaré, 2010).

## Author contributions

PC performed all experimental work. PC and SG did the data analysis. RS designed the research. All authors wrote the manuscript.

### Conflict of interest statement

The authors declare that the research was conducted in the absence of any commercial or financial relationships that could be construed as a potential conflict of interest.
