# Supplementary material for: Tb3+-Cleavage Assays Reveal Specific Mg2+ Binding Sites Necessary to Pre-fold the btuB Riboswitch for AdoCbl Binding
Source: Front Chem. 2017 Mar 21;5:10. doi: 10.3389/fchem.2017.00010 (PMC5359240; doi:10.3389/fchem.2017.00010)
Supplement: Supplementary file 1 [file Presentation1.PDF]

Supplementary Material

Tb^{3+}-cleavage assays reveal specific Mg^{2+} binding sites necessary to pre-fold the *btuB* riboswitch for AdoCbl binding

Pallavi K. Choudhary[1,2], Sofia Gallo[1] and Roland K. O. Sigel[1,*]

[1] Department of Chemistry, University of Zürich, Winterthurerstrasse 190, CH-8057 Zürich, Switzerland

[2] current address: Center for Molecular Biology of RNA, University of California at Santa Cruz, Santa Cruz, California 95064, USA

* corresponding author: roland.sigel@chem.uzh.ch

Supplementary Table

Suppl. Table 1: Contact map for the Mg^{2+} ions from the AdoCbl riboswitch of *S. thermophilum*. Shown are the MINAS predicted co-ordination for two Mg^{2+} ions, Mg (A310) and Mg (A311), reported in the crystal structure of the AdoCbl riboswitch (*S. thermophilum*) (PDB ID: 4GXY) (2).

	**Mg (A310)**	
**Bases**	**Atom**	**Distance (Å)**
G96	O6	5.197
G97	O6	3.352
	N7	5.25
G98	O6	3.546
	N7	5.432
G130	N7	4.534
	OP1	3.65
	OP2	4.874
	O4'	4.518
	**Mg (A311)**	
**Bases**	**Atom**	**Distance (Å)**
G65	O2'	4.252
U66	OP1	5.343

Supplementary Figures

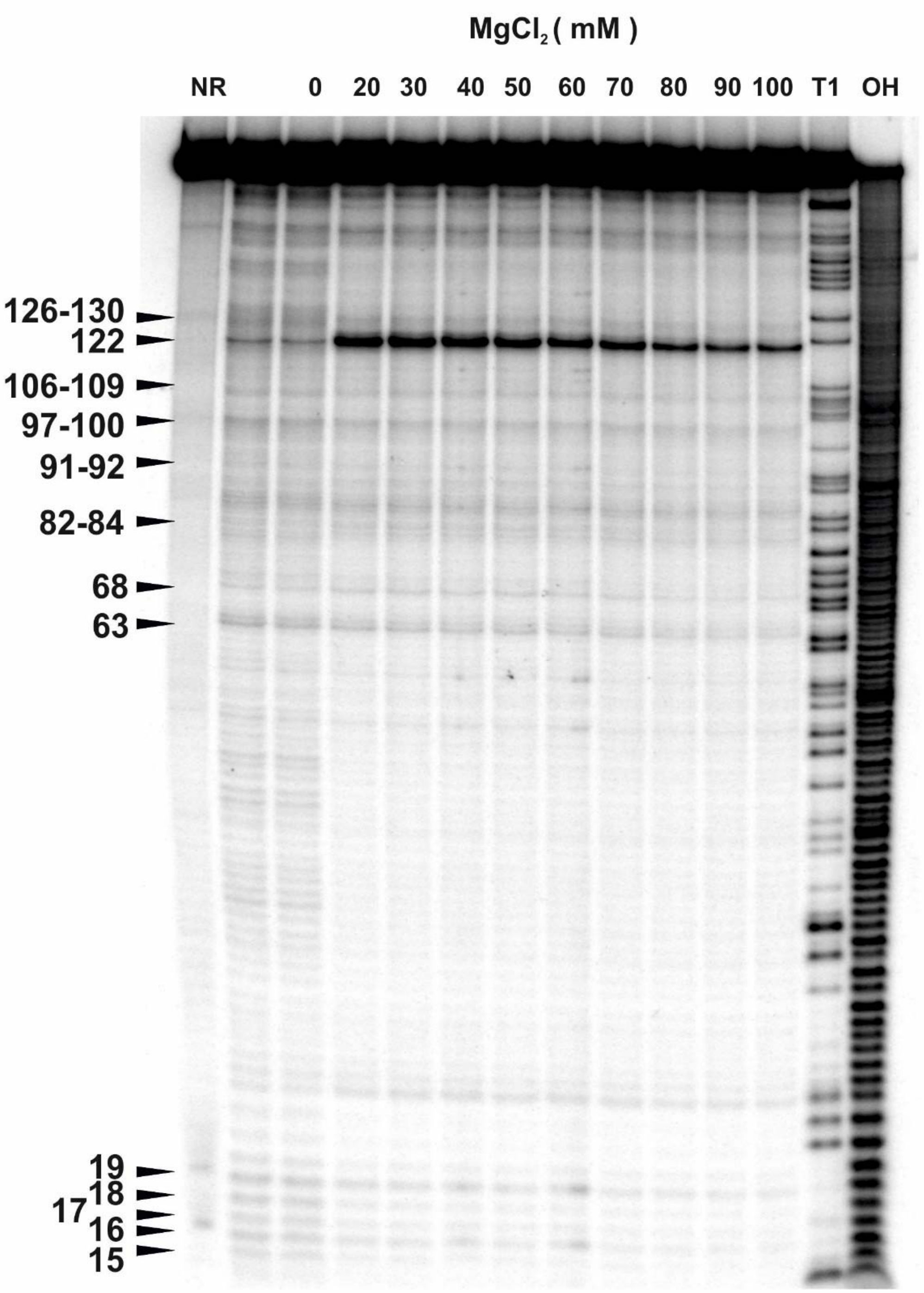

Suppl. Fig. 1: Competition experiment between Mg^{2+} and Tb^{3+} for the metal ion binding sites in the *btuB* aptamer. Nucleotides undergoing a distinct decrease in Tb^{3+} cleavage by 500 µM $TbCl_3$ are indicated. Mg^{2+} concentrations range from 0–100 mM. NR, non-reacted RNA; T1, RNase T1 ladder; OH, alkaline hydrolysis ladder.

(A)

(B)

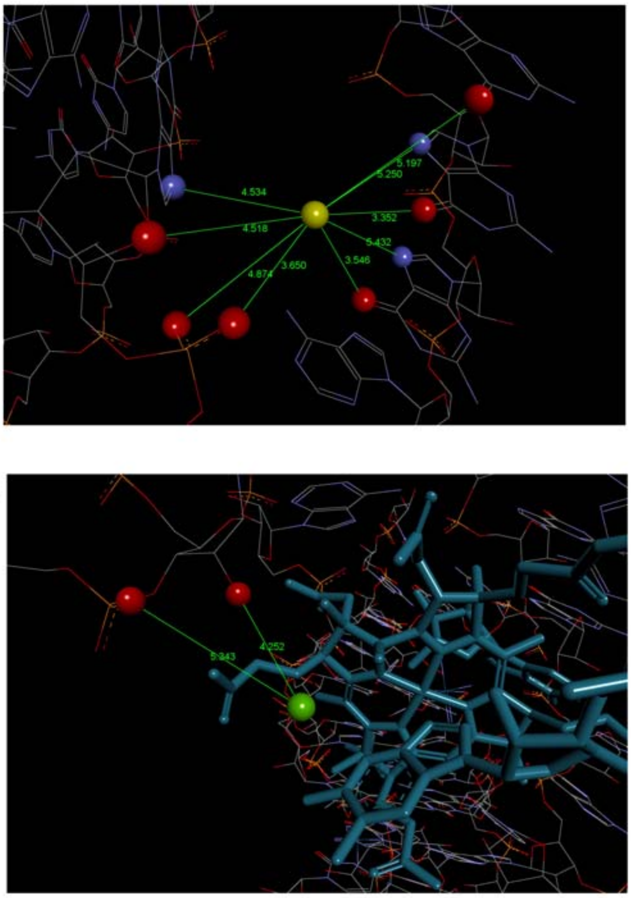

Suppl. Fig. 2: Mg^{2+} binding sites detected by MINAS (1) in the AdoCbl riboswitch of *S. thermophilum* (2). Shown are the bases predicted by MINAS contacting in an outer sphere manner to the two magnesium(II) ions, Mg A310 (**A**) and Mg A311 (**B**), in the crystal structure of the AdoCbl riboswitch from *S. thermophilum* (PDB ID: 4GXY).

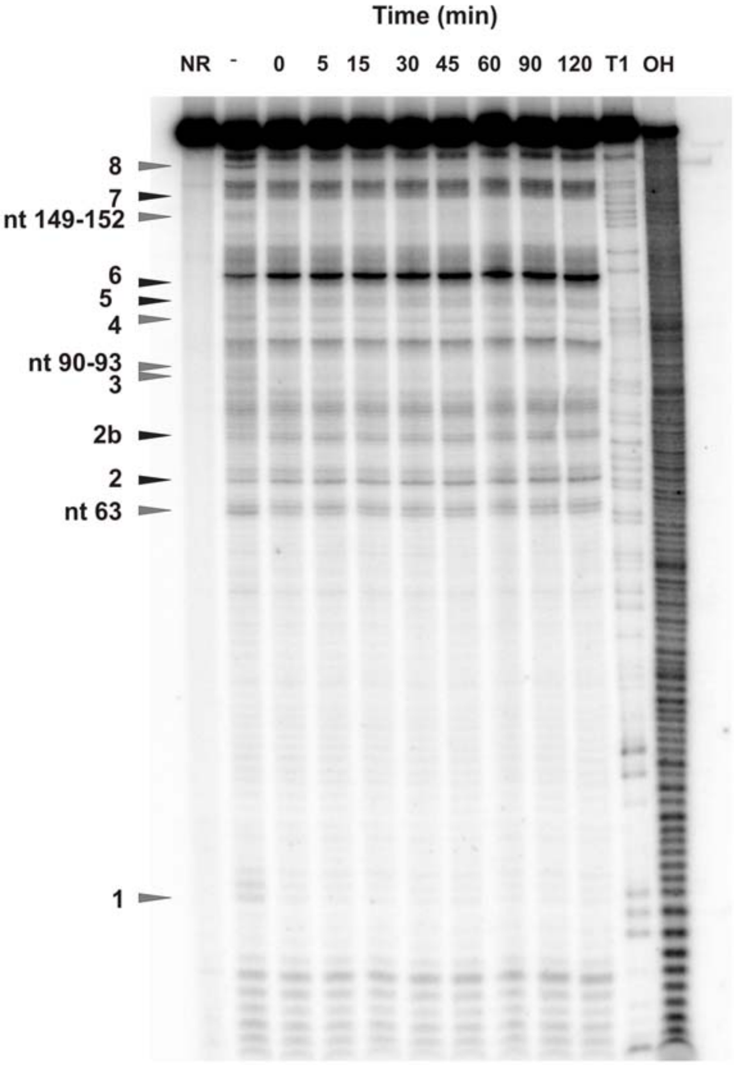

Supple. Fig. 3: Tb^{3+} cleavage on the AdoCbl bound *btuB* aptamer indicating an instantaneous structural switch. The prefolded *btuB* riboswitch was incubated with 100 µM AdoCbl for an indicated time period before the addition of 1mM $TbCl_3$. The grey and black arrows indicate the sites undergoing decrease and increase in the cleavage intensity upon incubation with AdoCbl respectively and represent similar pattern to the one observed in in-line probing experiments (3,4). Time of 0 minute implies an instant addition of 1 mM Tb^{3+} after the addition of AdoCbl. NR: non-reacted RNA, T1: RNase T1 ladder, OH: Alkaline hydrolysis ladder.

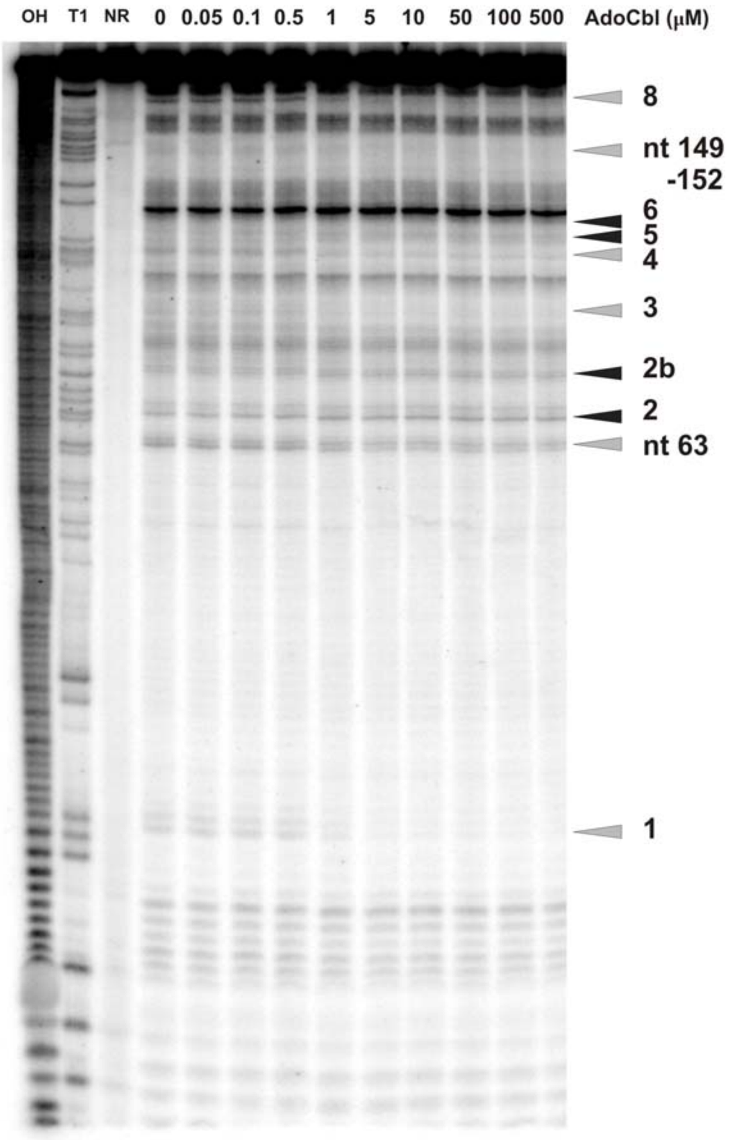

Suppl. Fig. 4: AdoCbl dependent Tb^{3+} cleavage of the *btuB* apatmer. The prefolded *btuB* aptamer was incubated with the indicated concentrations of AdoCbl for 30 minutes before the addition of 1 mM $TbCl_3$. The grey and black arrows indicate sites undergoing decrease and increase in the cleavage intensity upon incubation with AdoCbl. OH: Alkaline hydrolysis ladder, T1: RNase T1 ladder, NR: non-reacted RNA.

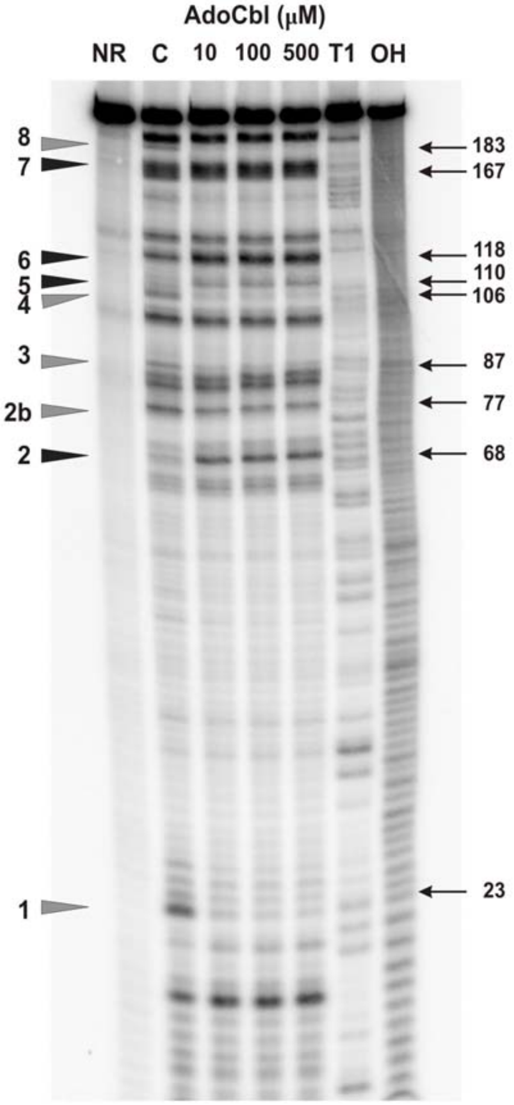

Suppl. Fig. 5: Switching of the modified 212 nucleotide long *btuB* aptamer in the presence of AdoCbl. The sites modulated by AdoCbl on the modified aptamer resemble that of the classical 202 nucleotide *btuB* aptamer. The black and the grey arrows on left indicate the sites undergoing decrease and increase in cleavage intensity respectively upon interaction with AdoCbl. The arrows at the right side indicate the corresponding position of the nucleotides undergoing AdoCbl induced changes. NR: No reaction, C: RNA incubated without AdoCbl, T1: RNase T1 ladder, OH: alkaline hydrolysis ladder. Note that the additional nucleotides at the 5'-end are excluded from the numbering scheme.

References

1. Schnabl, J., Suter, P. and Sigel, R.K.O. (2011) MINAS--a database of Metal Ions in Nucleic AcidS. *Nucleic Acids Res*, **40**, D434-438.
2. Peselis, A. and Serganov, A. (2012) Structural insights into ligand binding and gene expression control by an adenosylcobalamin riboswitch. *Nat Struct Mol Biol*, **19**, 1182-1184.
3. Gallo, S., Oberhuber, M., Sigel, R.K. and Krautler, B. (2008) The corrin moiety of coenzyme B12 is the determinant for switching the btuB riboswitch of E. coli. *Chembiochem*, **9**, 1408-1414.
4. Nahvi, A., Sudarsan, N., Ebert, M.S., Zou, X., Brown, K.L. and Breaker, R.R. (2002) Genetic control by a metabolite binding mRNA. *Chem Biol*, **9**, 1043-1049.
